# Spiritual Psychotherapy for Adolescents with Conduct Disorder: Designing and Piloting a Therapeutic Package 

**Published:** 2017-10

**Authors:** Mohammad Reza Mohammadi, Maryam Salmanian, Bagher Ghobari-Bonab, Jafar Bolhari

**Affiliations:** 1 Psychiatry and Psychology Research Center, Tehran University of Medical Sciences, Tehran, Iran.; 2 Psychology and Education of Exceptional Children Department, University of Tehran, Tehran, Iran.; 3 School of Behavioral Sciences and Mental Health, Iran University of Medical Sciences, Tehran, Iran.

**Keywords:** *Adolescents*, *Conduct Disorder*, *Object Attachment*, *Spiritual Therapie*

## Abstract

**Objective:** Spiritual psychotherapy has been conceptualized in the context of love and belief as principles of existence. Spiritual psychotherapy can provide an opportunity to design programs to treat conduct disorder. The aim of this study was to introduce the Spiritual Psychotherapy Package for Adolescents with Conduct Disorder and execute it as a pilot study.

**Method:** The intervention is a manual-guided program conducted over 14 group sessions, using the perspectives of object relations and attachment approach. It was executed for a group of eight adolescent boys with conduct disorder (mean age: 17.01 years) at Tehran reformatory. The Aggression Questionnaire and the Attachment to God Inventory were completed pre- and post-intervention.

**Results:** There were no significant differences in outcome measures from pre- to post- intervention. Cohen's dav was applied to estimate the measure of the effect size in this study. Cohen's dav measures of avoidance and anxious attachment to God showed acceptable effect sizes. However, Cohen's dav measure of verbal aggression indicated a small effect size.

**Conclusion:** We found evidence indicating acceptability of spiritual psychotherapy among adolescents with conduct disorder in attachment to God.

Conduct disorder is characterized by aggressive behaviors, deceitfulness or theft, destruction of property and serious violations of rules prior to age of 18 (1). The global burden of conduct disorder is considerable, particularly in males (2). The worldwide prevalence of conduct disorder has been reported as 3.6% for males and 1.5% for females (3). Mohammadi et al. (2014) reported a rate of 32.9% for conduct problems (DSM 5: 313.81) among Iranian children and adolescents (4).

existence: belief in the sacred, belief in unity, and belief in transformation; and love of others, love of work, and love of belonging (6).

Spiritual interventions that are independent of secular, evidence-based treatments for children and adolescents do not inherently depend on any particular theoretical orientation, but they have been adapted to treat children and adolescents on the basis of different theoretical perspectives (7).

A majority of studies found that antisocial behaviors such as delinquency and criminality were less prevalent among religious individuals (8). For example, Laird et al. (2011) found an association between low religious tendencies and high antisocial and rule-breaking behaviors among adolescents (9). Johnson (2003) reported less return to prison among those who participated in faith-based prison programs than among those in a control group (10).

In this study, we introduced a Spiritual Psychotherapy Package for Adolescents with Conduct Disorder as a manual-guided program to be executed in 14 group sessions, which employs the perspectives of object relations and attachment theories. Attachment and object relations theories provide a framework to explain interpersonal relationships (11) and delineate an integrative model to understand the problems of conduct disorder. The early relationship with primary caregiver forms expectations for the self and others, and influences the interpersonal interactions over the lifetime (12). An unavailable and rejecting caregiver can develop an avoidant-insecure attachment pattern in the child, which may result in conduct disorder (13). Several studies have reported avoidant and insecure attachment patterns among children and adolescents with conduct disorder (14-19). Nevertheless, there is a compensatory mechanism for insecure attachment; attachment to God can compensate for insecure human attachments (20). Overall, spiritual psychotherapy can play an important role in improving the attachment patterns of adolescents with conduct disorder. 

The aim of this study was to design and introduce the Spiritual Psychotherapy Package for Adolescents with Conduct Disorder and execute it as a pilot study. Based on previous research 12, 13, 20) we anticipated that, compared to the baseline, at study completion attachment to God would have increased and aggressive behavior decreased.

## Materials and Methods

We designed the Spiritual Psychotherapy Package for Adolescents with Conduct Disorder using the perspectives of object relations and attachment theories, as fourteen 90-minute sessions for groups of 6 to 8 adolescents with conduct disorder, aged 14 to 18 years, with or without substance use or attention deficit hyperactivity disorders; this package was introduced to the participants who were resident in reformatories. 

As demonstrated in Table 1, the 14 group therapy sessions of the Spiritual Psychotherapy Package for Adolescents with Conduct Disorder draw on principles of existence including belief in the sacred, belief in unity, and belief in transformation, love of others, love of work, and love of belonging (6); it also draws on spiritual interventions including acceptance, spiritual awareness, prayer, sacred texts, God images and forgiveness (7). These 14 sessions targeted impaired object relations in adolescents with conduct disorder including insecurity and fear, pessimism and mistrust, inability and abjection, and non-maintenance of boundaries and limits (21) and seeks to reform them. In the sessions, the stories, group discussions, allegories, verses of Quran, writing, poetry, meditation, painting, spiritual guided imagery, and prayer were used as practices (7, 22-31). The designer and deliverer of the package was a PhD by research candidate in clinical psychology, who had successfully completed psychoanalytic group therapy and spiritual psychotherapy courses at the Tehran University of Medical Sciences. The therapist who delivers the package should at least have a master’s degree in psychology, be interested and trained in spiritual psychotherapy, have adequate motivation to work with adolescents with conduct disorder, and should also complete 10 to 14 workshop sessions to learn to implement the package. 

The Spiritual Psychotherapy Package for Adolescents with Conduct Disorder was designed based on object relations of adolescents with conduct disorder (21) in focused sessions with experts. To assess the validity of the package, eight experts rated each session from 0 to 7 and provided their suggestions to improvement via a validity evaluation form. 

To test the feasibility of the package, it was delivered to a group of eight adolescent boys with conduct disorder aged 14 to 18 years, from February through March 2015 at Tehran reformatory. Inclusion criteria were: diagnosis of conduct disorder (DSM 5: 313.81); age 14 to 18 years; males, uninterrupted stay at a reformatory; written informed consent. Exclusion criteria were: acute suicidality; acute psychosis. The Aggression Questionnaire (32) and The Attachment to God Inventory (33) were completed before and after administration of the package. The study was approved by the Tehran University of Medical Sciences (Tehran, Iran) and conducted in accordance with the rules laid down in the Declaration of Helsinki. 

*Measures*

Aggression

The Aggression Questionnaire contains of 29 questions with four subscales covering physical aggression, verbal aggression, hostility, and anger; scores on the four subscales are summed to provide a total aggression score. These subscales have internal consistency over time (32). Mohammadi (2006) reported Cronbach’s alpha, retest, and split-half of 0.89, 0.78 and 0.73, respectively (34). In another study a reliability coefficient of 0.78 was reported for the Persian version of the Aggression Questionnaire (35).

*Attachment to God*

The Attachment to God Inventory is a 24-item scale that measures two subscales of avoidance and anxious attachment to God. Beck and McDonald (2004) reported factor eigenvalue of 6.51 and 3.88 for avoidance and anxious subscales, respectively (33). Olyaei et al. (2011) reported Cronbach’s alpha, and retest coefficients of 0.68, and 0.58 for the Persian version of the Attachment to God Inventory (36).

*Statistical Analysis*

Distributional data characteristics revealed normal distribution, and parametric tests were applied. Paired samples t-test was used to examine pre- to post-intervention differences using SPSS 16.0 (SPSS, Chicago IL, USA). Since Cohen's dav is used for within-subjects designs, we applied it to estimate effect sizes in this study.

## Results

Six out of the eight enrolled participants completed the intervention and all the study assessments; two participants were released from the reformatory and could not complete the program. The mean age of the participants was 17.1±0.75 years in this study. The mean years of education, and maternal and paternal education were 8.8, 8.4 and 6.8 years, respectively. Of the participants, four had a history of crime, four of their parents were divorced, five of them and their family members had substance use disorder, and three family members with criminal and psychiatric disorder histories. As Table 2 indicates there were no significant differences in outcome measures between pre- and post- treatment. There were decreases in scores on the avoidance and anxious attachment subscales of the Attachment to God Inventory from pre- to post-treatment. Cohen's dav measures for these two subscales showed acceptable effect sizes. However, an increase in the mean measures of the verbal aggression indicated an increase in the verbal aggression in the post-treatment as compared to the pre-treatment. Cohen's dav measure of verbal aggression indicated small effect size (Table 2).

**Figure 1:**
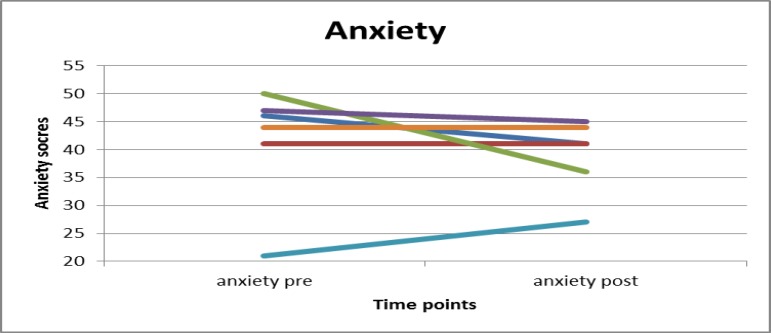
Changes of Anxiety scores from pre- to post-assessment. Anxiety scores of three participants decreased, remained stable in the case of two participants, and in the case of one participant worsened. Endpoints are means.

**Figure 2:**
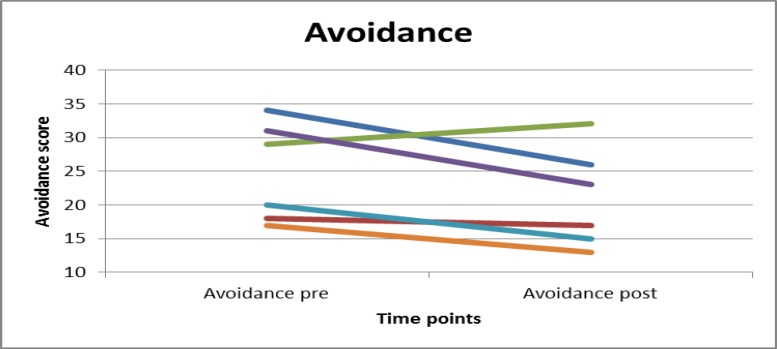
Individual changes of Avoidance scores from pre- to post-assessment. In five participants avoidance decreased; in one participant avoidance increased. Endpoints are means.

Figures 1 and 2 present the changes in anxiety and avoidance in graphical form. The graphs show that anxiety scores of three participants decreased, remained stable in the case of two participants, and in the case of one participant worsened. Avoidance score decreased for five participants, while for one participant it increased.

**Table 1:** Group Therapy Sessions of Spiritual Psychotherapy Package for Adolescents with Conduct Disorder

**Session**	**Title**	**Therapeutic goals**
1	Interview, Diagnosis, and Evaluation of Comorbidities	Using Kiddie Schedule for Affective Disorders and Schizophrenia for School-Age Children— Present and Lifetime (K-SADS-PL) to Diagnose Conduct Disorder and Comorbidities Included Substance Use and Attention-Deficit Hyperactivity (ADHD) Disorders, Evaluation IQ Using Raven's Progressive Matrices, and Completing Demographic Form
2	Acceptance and Build Trust	Providing an Overview of Spiritual Psychotherapy, and Increasing Trust and Security Targeting Object Relations based on Pessimism and Mistrust, and Insecurity and Fear Practice: Telling a Story, Setting up Group Discussions, Using the Allegory of Mirror, Meditation and Praying
3	Clarification and Reality Testing	Considering the Realities of Life, Clarification of Emotions, Thoughts and Behaviors Targeting Object Relations based on Pessimism and Mistrust, and Inability and Abjection Practice: Telling Stories, Setting up Group Discussions, Using the Allegory of Elastic Waistband and Praying
4	Assume Responsibility	Increasing Acceptance of Behaviors Responsibility and Providing Proper Understanding about Fate Targeting Object Relations based on Pessimism and Mistrust, Inability and Abjection, and Non-maintenance of Boundaries and Limits Practice: Telling Stories, Reading a Verse of the Quran, Setting up Group Discussions, Painting, and Praying
5	Self-awareness and Attachment	Modification of Attachment Patterns, Increasing Awareness of Self- Others Relations Targeting Object Relations based on Insecurity and Fear, Pessimism and Mistrust, Inability and Abjection, and Non-maintenance of Boundaries and Limits Practice: Reading a Part of a Sermon of Nahjolbalaghe, Using Spiritual Guided Imagery and Prayer
6	Flexibility, Satisfaction and Prayer	Increasing an Understanding of Personal Role in the Existent Problems, Moderating Expectations of God, and Increasing Flexibility to Change Problematic Behaviors Targeting Object Relations Based on Insecurity and Fear, and Non- maintenance of Boundaries and Limits Practice: Telling Stories, Reading Verses of the Quran, Setting up Group Discussions, Using the Allegories of Magnifying Glass and Sticking Plaster, Prayer and Meditation
7	Enhancing Love of Self, Others, Work, and Belonging	Increasing Awareness of Personal Assets and Paying Attention to Own Advantages, and Enhancing Generosity and Magnanimity Targeting Object Relations based on Pessimism and Mistrust, and Inability and Abjection Practice: Telling Stories, Setting up Group Discussions, Using Self Image, Writing, and Praying
8	Forgiveness	Increasing the Power of Forgiveness to Transgressor in Self-Other Relations, and Being Rescued from Hatred Targeting Object Relations based on Pessimism and Mistrust, and Insecurity and Fear Practice: Telling Stories and Proverbs, Setting up Group Discussions, Painting, Reading Verses of the Quran and Poetry, and Praying
9	Spiritual Awareness in Relation with Parents	Using Spiritual Mechanism to Compensate past Difficulties in Relation with Parents, and Increasing Acceptance of the Good and Bad Aspects of Parents as an Integrated Whole Targeting Object Relations based on Insecurity and Fear, and Pessimism and Mistrust Practice: Using Spiritual Imagery, Reading the Verses of Quran, and Praying
10	Spiritual Awareness and Belief in Unity	Increasing the Feeling of God Kindness, Decreasing Avoidant and Anxious Attachment to God Targeting Object Relations based on Insecurity and Fear, and Pessimism and Mistrust Practice: Telling Stories, Setting up Group Discussions, Reading Verses of the Quran, Using Spiritual Imagery and Praying
11	Spiritual Awareness and Belief in Transformation	Increasing Focus on the Future, and Thinking about Transformation at Life and after Death Targeting Object Relations based on Insecurity and Fear, Pessimism and Mistrust, Inability and Abjection, and Non-maintenance of Boundaries and Limits Practice: Telling Stories, Setting up Group Discussions, Reading Poetry, Using Spiritual Imagery, Painting, and Praying
12	Setting the Meaning and Purpose of Life	Increase in Selecting Reasonable, Realistic and Permanent Purposes, and Focusing on Goals Included with Positive aspects Targeting Object Relations based on Insecurity and Fear, Inability and Abjection, and Non-maintenance of Boundaries and Limits Practice: Telling Stories, Setting up Group Discussions, Using Spiritual Imagery, Painting, Writing and Praying
13	Review of the Previous Sessions	Providing Better Understanding about the Meaning and the purpose of the Previous Sessions Targeting Object Relations based on Insecurity and Fear, Pessimism and Mistrust, Inability and Abjection, and Non-maintenance of Boundaries and Limits Practice: Setup Group Discussions, Painting, Using the Allegory of Alarm Clock, Praying, and Meditating
14	Preparing for the End and Prevent Relapse	Increasing the Maintenance of the Treatment Results and Preventing Recurrence, and Selecting a Guide Included Positive Traits Targeting Object Relations based on Insecurity and Fear, Inability and Abjection, and Non-maintenance of Boundaries and Limits Practice: Telling Stories, Setting up Group Discussions, Painting, Using the Allegory of Flashlight and Praying

**Table 2:** Paired Samples t-test Analysis between Pre-Post Treatments and Cohen's dav Estimation

**Outcome Measures**	**t**	**Sig**	**Mean (SD) ** **Pre-treatment**	**Mean (SD) ** **Post-** **treatment**	**Mean ** **Difference**	**Mean ** **of SDs**	**Cohen** ***'s *** ***d***_av_
Attachment to God Inventory							
Subscales	2.203	0.079	24.83 (7.35)	21 (7.29)	3.8	7.32	0.51
Avoidance Attachment							
Anxious Attachment	0.916	0.402	41.5 (10.48)	39 (6.66)	2.5	8.57	0.29
Aggression Questionnaire							
Subscales	0.000	1.00	30.83 (6.21)	30.83 (4.87)	0	5.54	0
Physical Aggression	-1.472	0.201	15.66 (2.50)	16.83 (1.94)	-1.167	2.22	-0.52
Verbal Aggression	0.000	1.00	26.50 (6.97)	26.50 (7.23)	0	7.10	0
Hostility	-0.326	0.758	23.33 (3.61)	23.66 (4.58)	-0.33	4.10	-0.08
Anger							
Total Aggression	-0.288	0.785	96.33 (17.03)	97.83 (16.43)	-1.5	16.73	-0.08

## Discussion

This is the first study to design and introduce the Spiritual Psychotherapy Package for Adolescents with Conduct Disorder and conduct it as a pilot study to investigate the effects of the treatment on participants’ attachment to God. Because of the small sample of the pilot study, no significant differences in outcome measures were found between pre- post-treatment. 

However, acceptable effect sizes were observed with respect to avoidance and anxious attachment to God, indicating that these could be reduced by this intervention. Although verbal aggression had increased following treatment, this may be due to the intimate environment of the sessions, which enabled expression of verbal criticism rather than physical aggression. 

Arguments have been made for the benefits of various psychological interventions in reducing the symptoms of conduct disorder, including family and parenting interventions, multi-systemic therapy, parent management training, functional family therapy, various behavioral management strategies, multidimensional treatment foster care, and cognitive-behavior skills building programs (37-39). Nonetheless, they have had limited effectiveness in the treatment of conduct disorder, particularly among adolescents where the problems have been severe and associated with seriously disturbed families (5).

A majority of studies have shown religiosity and spirituality to have significant effects on delinquent and antisocial behaviors (8, 9, 40, 44). No significant effect was observed in the intervention, however, if the intervention introduced in this study were to be tested with conduct disordered adolescents in clinical trial designs this may confirm the findings of the previous research that has used spiritual interventions with delinquent groups. For example, Johnson (2003) reported less return to prison among those prisoners who participated in a faith-based prison program as compared to a control group. Hausmann and Spooner (2009) provided pastoral counseling interventions for 16 adolescent male delinquents and found such counseling to be a contributing factor in successful treatment and low recidivism (45).

In addition, several studies have reported avoidant and insecure attachment patterns among children and adolescents with conduct disorder (14-19), and have also found that attachment to God can compensate for insecure human attachments (20). Having designed the Spiritual Psychotherapy Package for Adolescents with Conduct Disorder the pattern of results suggested that the intervention had a favorable influence. Therefore, we suggest that this package be implemented in clinical trials with control group. 

## Conclusion

We introduced the Spiritual Psychotherapy Package for Adolescents with Conduct Disorder and executed it as a pilot study. 

Because of the small sample of the pilot study, no significant differences in outcome measures were found between pre- post-treatment. However, we found evidence indicating acceptability of spiritual psychotherapy among adolescents with conduct disorder in attachment to God.

## Limitation

This study had the following limitations: 1) participants in this study were from one reformatory in Iran and due to the small sample size, the results of this study are not representative of all Iranian adolescents with conduct disorder; 2) lack of control group; 3) lack of follow-up; 4) No experts’ ratings. 
